# Occupational Therapists’ Psychotherapy Competence: A Scoping Review of Secondary Data

**DOI:** 10.1177/00084174251319768

**Published:** 2025-02-17

**Authors:** Andrea Mandzuk, Pamela Wener

**Keywords:** Mental health, Professional competence, Compétence professionnelle, santé mentale

## Abstract

**Background.** Occupational therapists have been writing about and practicing psychotherapy for almost a century. However, questions about competence and tensions regarding psychotherapy in occupational therapy persist both within and outside the profession. **Purpose.** To explore the scope of the existing literature on psychotherapy competence written by occupational therapists and/or pertaining to occupational therapy research or practice. **Method.** A secondary analysis of the 207 articles included in the scoping review by Marshall and colleagues was conducted. Using inductive and deductive approaches, data from 207 articles were screened, extracted, and analyzed to identify themes related to competence in psychotherapy. **Findings.** The 104 articles included spanned from 1927 to 2020; 50% were non-empirical. The narrative synthesis had one overall theme, Professional Identity, and three subthemes: Competence, Attaining and Maintaining Competence, and The Great Debate. There was no consistent pathway outlined for occupational therapists to attain psychotherapy competence, which may contribute to role confusion and dissonance. **Conclusion.** This review revealed the reciprocal relationship between professional identity and psychotherapy competence in occupational therapists. Future research should explore how the use of psychotherapy competence pathways impacts professional identity and contributes to practice competence.

## Résumé

**Description**. Les ergothérapeutes pratiquent la psychothérapie et écrivent à ce sujet depuis près d’un siècle. Cependant, les questions de compétence et les tensions en cette matière persistent tant à l’intérieur qu’à l’extérieur de la profession. **But.** Explorer la portée de la littérature existante sur la compétence en psychothérapie rédigée par des ergothérapeutes et/ou se rapportant à la pratique ou à la recherche en ergothérapie. **Méthodologie.** Une analyse secondaire des 207 articles inclus dans l’étude de portée de Marshall et ses collègues (2022) a été effectuée. À l’aide d’approches inductives et déductives, les données de ces 207 articles ont été passées au crible, extraites et analysées afin d’identifier des thèmes liés à la compétence en psychothérapie. **Résultats.** Les 104 articles inclus couvrent la période de 1927 à 2020; 50% d’entre eux sont non empiriques. La synthèse narrative a fait ressortir un thème général, l’identité professionnelle, et trois sous-thèmes : la compétence, atteindre et maintenir la compétence, et le Grand débat. Il n’en ressort pas de parcours cohérent pour permettre aux ergothérapeutes d’atteindre la compétence en psychothérapie, ce qui pourrait contribuer à la dissonance et à la confusion des rôles. **Conclusion.** Cette étude révèle la relation réciproque entre l’identité professionnelle et la compétence en psychothérapie chez les ergothérapeutes. Les recherches futures devraient explorer comment l’utilisation de parcours clairs pour atteindre la compétence en psychothérapie influe sur l’identité professionnelle et contribue à la compétence pratique.

## Introduction

The demand for mental health services including psychotherapy has significantly increased in recent years (Moroz et al., 2020; World Health Organization, 2022), and those seeking services experience long wait times due to a shortage of professionals (Moroz et al., 2020). Occupational therapists are one group of professionals who integrate psychotherapy as part of their practice (Marshall et al., 2022; Moll et al., 2013).

Psychotherapy is an “…interpersonal treatment…based on psychological principles; [delivered by] a trained therapist [to] a client who is seeking help for a mental disorder, problem, or complaint… [and] is adapted or individualized for the particular client…” (Wampold & Imel, 2015, p. 37). Occupational therapists have an occupation-based approach to psychotherapy, using meaningful activities (occupations) to achieve improved mental health and well-being (Gillen & Brown, 2024; Marshall et al., 2022; Wilcock, 1999). By integrating models like the Person-Environment-Occupation (PEO) model, occupational therapists tailor therapeutic approaches to each client's daily life and environment (Law et al., 1996). Common psychotherapies used by occupational therapists include cognitive-behavioural therapy (CBT), motivational interviewing, mindfulness meditation, solution-focused therapy, and dialectical-behavioural therapy, each adapted to suit an occupation-centered perspective (Moll et al., 2013). Despite this unique approach and potential to increase access to psychotherapy, occupational therapists remain underrecognized as psychotherapy providers (Henderson et al., 2015; Moll et al., 2013).

In Canada, psychotherapy is governed provincially. In some provinces such as Quebec, occupational therapists practicing psychotherapy require an additional permit, while in other provinces such as Manitoba, psychotherapy is not regulated beyond the general professional regulations. In Ontario, occupational therapists practicing psychotherapy are regulated by the College of Occupational Therapists of Ontario (COTO) but have standards specifically governing psychotherapy practice (COTO, 2018, 2023).

Competencies are a combination of “the knowledge, skills, and attitudes that are required for an occupational therapist to practice safely, effectively, and ethically” (College of Occupational Therapists of British Columbia, n.d., para. 3). Knowledge refers to the theoretical understanding necessary for competent practice (Kaslow, 2004). Skills refer to the ability to apply knowledge in practice (Kaslow, 2004). Attitudes include the mindsets and dispositions that shape a professional's approach to their work (Fouad et al., 2009; Kaslow, 2004; Sperry, 2022). Competence refers to one's ability to demonstrate competencies (Association of Canadian Occupational Therapy Regulatory Organizations [ACOTRO] et al., 2021).

As a self-regulated profession, occupational therapists must self-assess their competence (COTM, 2010), but the absence of psychotherapy-specific guidelines can leave providers uncertain about their abilities (Moll et al., 2013). For some therapists, the lack of competencies or standards leaves them questioning the role of psychotherapy in occupational therapy practice (Moll et al., 2013). Moreover, being unsure of one's competence and knowledge impacts occupational therapists’ professional identity (Walder et al., 2022). Professional identity is an individual's sense of belonging to their professional group, which shapes their attitudes, values, and behaviors in practice (Abreu, 2006; Beauchamp & Thomas, 2009; Chreim et al., 2007; Dige, 2009; Fitzgerald, 2020; Kreiner et al., 2006; Schein, 1978).

This paper is an initial step in the exploration of psychotherapy competence in the occupational therapy peer-reviewed literature. A recent scoping review by Marshall and colleagues (2022) examined 207 articles from the occupational therapy literature to explore psychotherapy in the occupational therapy literature. The current study examined the same 207 articles to explore psychotherapy competence in the peer-reviewed occupational therapy literature. The research question for this study is as follows: What is the scope of existing literature on psychotherapy competence within the occupational therapy literature and/or pertaining to occupational therapy research or practice?

## Method

This scoping review used secondary data analysis to explore psychotherapy competence within occupational therapy, narrowing the focus of the original review by Marshall et al. (2022). In secondary data analysis, the researcher asks new and related questions about data collected for another purpose (Cheng & Phillips, 2014; Glass, 1976; Heaton, 2011; Smith, 2011; Wickham, 2019). This study followed the Arksey and O’Malley framework (Arksey & O’Malley, 2005) and was guided by PRISMA-ScR (Tricco et al., 2018), and the JBI Manual for Evidence Synthesis (Peters et al., 2020).

Although less common in scoping reviews, secondary analysis uses existing datasets to explore new questions (Hakim, 1982; Heaton, 2011). In this study, the dataset consisted of articles from Marshall and colleagues (2022), requiring a clear alignment between the original data and the current research question. In secondary analysis, the researcher must have a thorough understanding of the original data set and the topic area, to ensure that the research question and purpose of the secondary analysis is within the parameters of, and aligns with, the original data set (Thorne, 1998; Wickham, 2019). The second author (PW) co-authored the original scoping review, ensuring alignment with the original study.

This secondary analysis narrows the focus of the original scoping review to explore psychotherapy competence, within the broader context of psychotherapy within occupational therapy. By narrowing the scope of the original scoping review, this secondary analysis allows for a more narrow and deeper synthesis of evidence related to the psychotherapy competence of occupational therapists. Although this approach is not widely discussed in the scoping review literature, it follows the principles of secondary analysis (Cheng & Phillips, 2014; Heaton, 2011; Thorne, 1998; Wickham, 2019), where pre-existing data, i.e., the articles included in the original scoping review, are re-analyzed for a different purpose. The authors determined that the original data set fit well with the purpose of this secondary analysis (Thorne, 1998; Wickham, 2019). By exploring the competence of occupational therapists in providing psychotherapy, the secondary analysis remains anchored within the broader topic of psychotherapy in occupational therapy.

### Article Selection

The principal investigator of the original study provided 207 articles for the current study. Details of the search strategy and inclusion and exclusion criteria for the 207 articles are available in the study by Marshall and colleagues (2022). The 207 articles were uploaded into Covidence, a web-based program used for screening. During title and abstract review, competence-related terms were absent, so a full-text screen was conducted using the inclusion and exclusion criteria listed in Table 1. Terms related to psychotherapy competence of occupational therapists were based on definitions of competence and competencies cited in the introduction. Terms included competence, competency, competencies, training/education, knowledge, skills, attitudes, regulation, governance, and guidelines. In addition to searching for these terms, the researchers also scanned for other content related to competence. Disagreements regarding article inclusion were discussed and consensus was reached at weekly research meetings.

**Table 1:** Inclusion and Exclusion Criteria

Inclusion Criteria	Exclusion Criteria
1. Articles pertaining to psychotherapy competency of occupational therapists that were published in an occupational therapy journal or include an author who identifies as an occupational therapist	1. Non-peer-reviewed sources (e.g., book reviews, books, anecdotal reports, and commentary)
2. Dissertations and theses	2. Conference abstracts.
3. Articles published in all years	3. Articles exploring mental health approaches that were inconsistent with the definition of psychotherapy used in this study
4. Articles pertaining to all age groups	4. Articles that explored interventions that did not involve a component of discussion and psychological processing
5. Articles published in English	5. Articles exploring solely psychoeducational or independent living skills training

### Data Analysis

Data from the included articles were extracted by the first author (AM) and reviewed by the second author (PW), including author(s), title, study design, country, and findings related to the research question. The analysis used both deductive (pre-identified competence definitions) and inductive (emerging codes and categories) approaches. Inductive content analysis is an appropriate method of data analysis when there is little research published in the area (Kyngäs, 2020), such as in the case of psychotherapy competence of occupational therapists.

The analysis process began with open coding, where the first researcher assigned descriptive codes to data related to competence. Codes were compared and grouped together to create broader categories or themes (Kyngäs, 2020; Vears & Gillam, 2022). This was an iterative process that involved continuous discussion and refinement of codes and categories (Vears & Gillam, 2022).

Interpretation is inherent in secondary data analysis (Thorne, 1998; Vears & Gillam, 2022). In this study, interpretation was used for understanding and articulating nuanced insights about competence in psychotherapy within occupational therapy. The researchers carefully interpreted the data to maintain its original meaning, supported by the second author's involvement in the original study. Some data, such as general mentions of training, were broad. Interpretation helped clarify competence as a dynamic, multifaceted construct influenced by specific and broad references.

## Results

The scope of the existing literature on psychotherapy competence within occupational therapy includes the elements of psychotherapy competence, attainment and maintenance, the debate surrounding psychotherapy within occupational therapy and its impacts on professional identity.

### Descriptive Summary

**Figure 1.**
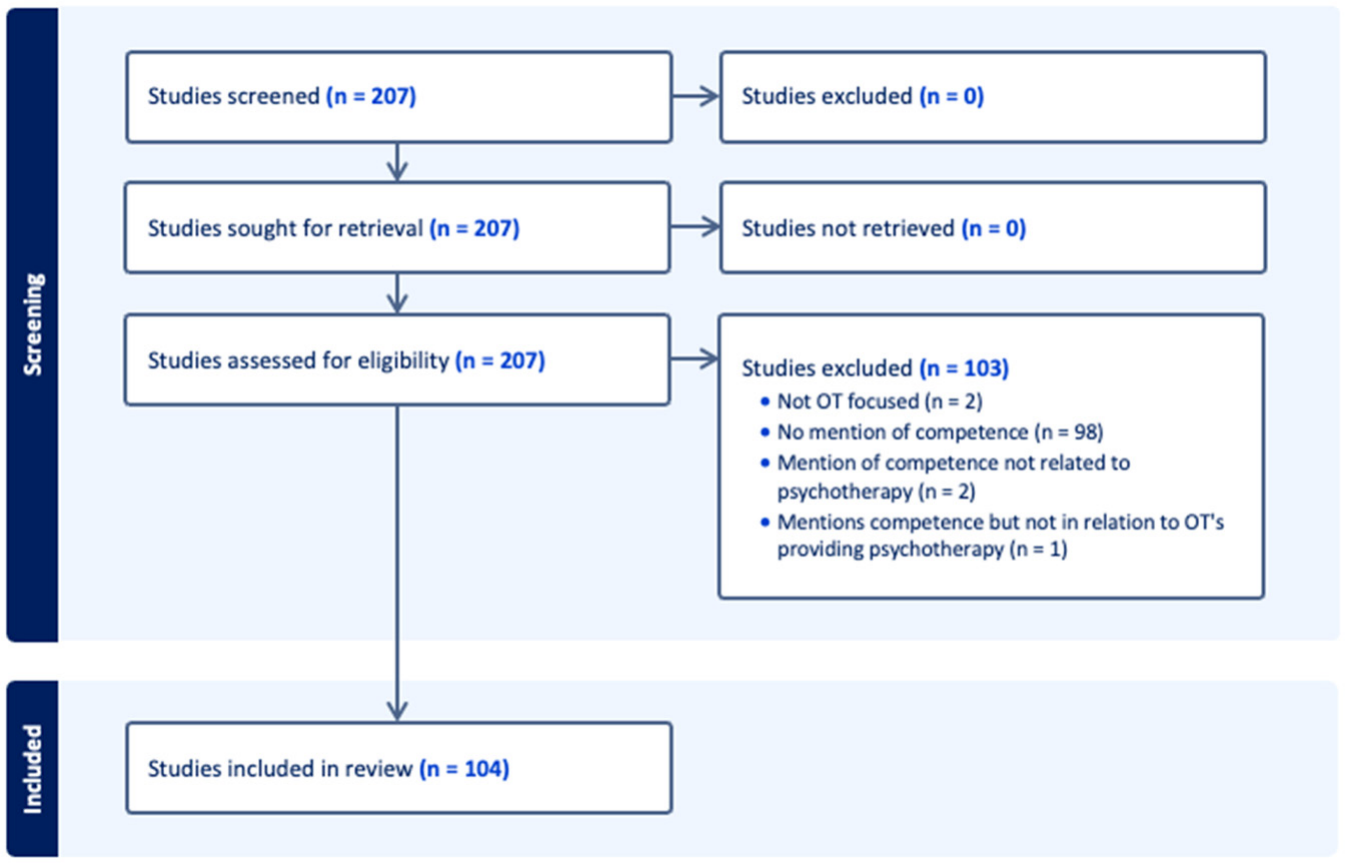
PRISMA diagram.

Over 50% of the 207 articles met the inclusion criteria (*n = *104; Table 2). The reasons for exclusion are detailed in Figure 1.

**Table 2:** List of Included Articles

1. Alers, V. (2008). The 20th Vona du Toit Memorial Lecture 2007: Proposing the social atom of occupational therapy: Dealing with trauma as part of an integrated inclusive intervention. *South African Journal of Occupational Therapy, 38(*3), 3–10.
2. Azima, H., & Wittkower, E. D. (1957). A partial field survey of psychiatric occupational therapy. *American Journal of Occupational Therapy, 24*(3), 69–80. doi: 10.1177/000841745702400302
3. Bailey, D. (1971). Occupational therapy in a crisis intervention unit of large general hospital. *Australian Journal of Occupational Therapy, 18*(4), 13–17. doi: 10.1111/j.1440–1630.1971.tb00499.x
4. Banks, E., & Blair, S. E. E. (1997). The contribution of occupational therapy within the context of the psychodynamic approach for older clients who have mental health problems. *Health Care in Later Life, 2*(2), 85–92.
5. Birkholtz, M., & Blair, S. E. (2001). Chronic pain: the need for an eclectic approach part 2. *British Journal of Therapy and Rehabilitation, 8(*3), 96–103. doi: 10.12968/bjtr.2001.8.3.13741
6. Bostick, G. P. (2017). Effectiveness of psychological interventions delivered by non-psychologists on low back pain and disability: a qualitative systematic review. *The Spine Journal, 17*(11), 1722–1728. doi: 10.1016/j.spinee.2017.07.006
7. Brick, R., Lyons, K. D., Rodakowski, J., & Skidmore, E. (2020). A Need to Activate Lasting Engagement. *American Journal of Occupational Therapy, 74*(5), 7405347010p1-7405347010p5. https://doi.org/10.5014/ajot.2020.039339
8. Broadbent, F. (1985). Counselling: an introduction. *British Journal of Occupational Therapy, 48*(2), 36–39. doi: 10.1177/030802268504800203
9. Burton, A. (1954). The occupational therapist as therapist. *American Journal of Occupational Therapy, 8*(2), 78–79.
10. Carpenter, B. D. (2014). Contemporary psychological approaches to life at the end of life. *Occupational Therapy in Health Care, 28*(1), 31–41. doi: 10.3109/07380577.2013.867090
11. Champernowne, H. I. (1952). Art in occupational therapy as an inner remedial process in psychoneurotic and mental disorders*. British Journal of Physical Medicine: Including its Application to Industry, 15*(12), 281–290.
12. Chan, C., Dennis, D., Kim, S. J., & Jankowski, J. (2017). An integrative review of school-based mental health interventions for elementary students: Implictaions for occupational therapy. *Occupational Therapy in Mental Health, 33*(1), 81–101. https://doi.org/10.1080/ 0164212x.2016.1202804
13. Clark, J., & Robertshaw, S. (1990). Intensive group psychotherapy using a team of therapists I: Good for patients, good for staff?. *Group Analysis, 23*(2), 129–135. doi: 10.1177/0533316490232003
14. Clarke, C. (1999). Treating post-traumatic stress disorder: occupational therapist or counsellor? *British Journal of Occupational Therapy, 62*(3), 136–138. doi: 10.1177/030802269906200313
15. Cline, D. W., & Rouzer, D. L. (1971). The nonphysician as primary therapist in hospital psychiatry. *American Journal of Psychiatry, 128*(4), 407–411. doi: 10.1176/ajp.128.4.407
16. Clouston, T. (2003). Narrative methods: Talk, listening and representation. *British Journal of Occupational Therapy, 66*(4), 136–142. doi: 10.1177/030802260306600402
17. Cobbley, J. D. (1999). Reminiscence and life review use in occupational therapy. (Publication No. 1395596) [Master's thesis, Rush University]. *ProQuest Dissertations Publishing.*
18. Colman, W. (1975). Occupational therapy and child abuse. *American Journal of Occupational Therapy, 29*(7), 412–417.
19. Conlin, M., & Lorinser, A. (2012). The most effective evidence-based occupational therapy interventions for adolescents with bipolar disorder: A systematic literature review (Publication No. 1530611) the college of st. Scholastica*. ProQuest Dissertations Publishing.*
20. Cooper, P. R., & Davis, D. A. (2016). Using writing as therapy: finding identity—An evaluation of its effects upon clinical outcomes and service. *International Journal of Therapy and Rehabilitation, 23*(2), 64–74. https://doi.org/10.12968/ijtr.2016.23.2.64
21. Copley, B., Forryan, B., & O’Neill, L. (1987). Play therapy and counselling work with children. *British Journal of Occupational Therapy, 50*(12), 413–416. https://doi.org/10.1177/030802268705001205
22. Carlson, L., & Coyne, E. (2013). Effective occupational therapy interventions addressing rest and sleep for adults with Parkinson's disease (Publication No. 1552905) [Doctoral dissertation, The College of St. Scholastica]. *ProQuest Dissertations Publishing.*
23. Craik, C., Chacksfield, J. D., & Richards, G. (1998). A survey of occupational therapy practitioners in mental health. *British Journal of Occupational Therapy, 61*(5), 227–234. doi: 10.1177/030802269806100513
24. Davis, S. F., & Hyde, P. (2002). Priorities in mental health research: An update. *British Journal of Occupational Therapy, 65*(8), 387–389. doi: 10.1177/030802260206500807
25. DeCarlo, J. J., & Mann, W. C. (1985). The effectiveness of verbal versus activity groups in improving self–perceptions of interpersonal communication skills. *American Journal of Occupational Therapy, 39*(1), 20–27. doi: 10.5014/ajot.39.1.20
26. Eakman, A. M., Schmid, A. A., Henry, K. L., Rolle, N. R., Schelly, C., Pott, C. E., & Burns, J. E. (2017). Restoring effective sleep tranquility (REST): A feasibility and pilot study. *British Journal of Occupational Therapy, 80(*6), 350–360. doi: 10.1177/0308022617691538
27. Fisher, A., & Savin–Baden, M. (2001). The benefits to young people experiencing psychosis, and their families, of an early intervention programme: Evaluating a service from the consumers’ and the providers’ perspectives. *British Journal of Occupational Therapy, 64*(2), 58–65. doi: 10.1177/030802260106400202
28. Franca, R. D., & Milbourn, B. (2015). A meta-analysis of mindfulness based interventions (MBIS) show that MBIs are effective in reducing acute symptoms of depression but not anxiety. *Australian Occupational Therapy Journal, 62*(2), 147–148. doi: 0.1111/1440-1630.12198
29. Fraser, K., MacKenzie, D., & Versnel, J. (2019). What is the current state of occupational therapy practice with children and adolescents with complex trauma?. *Occupational Therapy in Mental Health, 35*(4), 317–338. https://doi.org/10.1080/0164212X.2019.1652132
30. Froehlich, J. (1992). Occupational therapy interventions with survivors of sexual abuse. *Occupational Therapy in Health Care, 8*(2–3), 1–25. doi: 10.1080/J003v08n02_01
31. Gagnon, D. L. (1996). A review of reality orientation (RO), validation therapy (VT), and reminiscence therapy (RT) with the Alzheimer's client. *Physical & Occupational Therapy in Geriatrics, 14*(2), 61–77. doi: 10.1080/J148v14n02_05
32. Gardiner, P., MacGregor, L., Carson, A., & Stone, J. (2017). Occupational therapy for functional neurological disorders: a scoping review and agenda for research. *CNS Spectrums, 23*(03), 205–212. doi:10.1017/S1092852917000797
33. Gerardi, S. M. (2017). Development of a consensus-based occupational therapy treatment template for veterans with combat-related post traumatic stress disorder: a delphi study (Publication No. 10287602) [Doctoral dissertation, Texas Woman's University]. *ProQuest Dissertations Publishing.*
34. German, S. A. (1964). A group approach to rehabilitation occupational therapy in a psychiatric setting*. American Journal of Occupational Therapy, 18*(5), 209–214.
35. Giles, G. M., & Chng, C. L. (1984). Occupational therapy in the treatment of anorexia nervosa: a contractual-coping approach. *British Journal of Occupational Therapy, 47*(5), 138–141. doi: 10.1177/030802268404700503
36. Gonzalez–Dolginko, E. (2008). The secret lives of art therapists: An exploratory study about the nature of the work that art therapists do in schools (Publication No. 3317750) [Doctoral dissertation, Hofstra University]. *ProQuest Dissertations Publishing.*
37. Green, A., Hicks, J., Weekes, R., & Wilson, S. (2005). A cognitive-behavioural group intervention for people with chronic insomnia: an initial evaluation. *British Journal of Occupational Therapy, 68*(11), 518–522. https://doi.org/10.1177/030802260506801106
38. Green, A., Hicks, J., & Wilson, S. (2008). The experience of poor sleep and its consequences: A qualitative study involving people referred for cognitive-behavioural management of chronic insomnia. *British Journal of Occupational Therapy, 71*(5), 196–204. https://doi.org/10.1177/030802260807100506
39. Griffiths, S. (2008). The experience of creative activity as a treatment medium*. Journal of Mental Health, 17*(1), 49–63. doi: 10.1080/09638230701506242
40. Griffiths, S., & Corr, S. (2007). The use of creative activities with people with mental health problems: A survey of occupational therapists. *British Journal of Occupational Therapy, 70*(3), 107–114. doi: 10.1177/030802260707000303
41. Gunnarsson, A. B., Jansson, J. A., & Eklund, M. (2006). The Tree Theme Method in psychosocial occupational therapy: A case study. *Scandinavian Journal of Occupational Therapy, 13*(4), 229–240. doi: 10.1080/11038120600772908
42. Gunnarsson, A. B., & Eklund, M. (2009). The tree theme method as an intervention in psychosocial occupational therapy: client acceptability and outcomes*. Australian Occupational Therapy Journal, 56*(3), 167–176. doi: 10.1111/j.1440-1630.2008.00738.x
43. Gunnarsson, A. B., Peterson, K., Leufstadius, C., Jansson, J. A., & Eklund, M. (2010). Client perceptions of the Tree Theme Method^TM^: a structured intervention based on storytelling and creative activities. *Scandinavian Journal of Occupational Therapy, 17*(3), 200–208. doi: 10.3109/11038120903045366
44. Gunnarsson, A. B., Jansson, J. A., Petersson, K., & Eklund, M. (2011). Occupational therapists’ perception of the tree theme method as an intervention in psychosocial occupational therapy. *Occupational Therapy in Mental Health, 27*(1), 36–49. doi: 10.1080/0164212X.2011.543630
45. Gunnarsson, A. B., Wagman, P., Hakansson, C., & Hedin, K. (2015). The tree theme method(R) (TTM), an occupational therapy intervention for treating depression and anxiety: Study protocol of a randomized controlled trial. *BMC Psychology, 3*(40), 1–7. https://doi. org/10.1186/s40359-015-0097-9
46. Gunnarsson, B., Wagman, P., Hedin, K., & Håkansson, C. (2018). Treatment of depression and/or anxiety–outcomes of a randomised controlled trial of the tree theme method^®^ versus regular occupational therapy. *BMC Psychology, 6*(1), 25. doi: 10.1186/s40359-018-0237-0
47. Haviland, C. F. (1927). Occupational therapy from the viewpoint of the superintendent of a state mental hospital*. American Journal of Physical Medicine & Rehabilitation, 6*(6), 431–438.
48. Hayner, K. A. (1999). Awareness of psychological issues and actual practices by occupational therapists evaluating and treating patients with selected physical disabilities (Publication No. 9933314) [Doctoral dissertation, University of San Francisco]. *ProQuest Dissertations Publishing.*
49. Hersche, R., Weise, A., Michel, G., Kesselring, J., Barbero, M., & Kool, J. (2019). Development and Preliminary Evaluation of a 3-Week Inpatient Energy Management Education Program for People with Multiple Sclerosis–Related Fatigue*. International Journal of MS Care, 21*(6), 265–274. DOI: 10.7224/1537-2073.2018-058
50. Hewlett, S., Ambler, N., Almeida, C., Blair, P. S., Choy, E., Dures, E., Rooke, C., Thorn, J., Tomkinson, K., & Pollock, J. (2015). Protocol for a randomised controlled trial for Reducing Arthritis Fatigue by clinical Teams (RAFT) using cognitive–behavioural approaches. *BMJ Open, 5*(8), e009061. doi: 10.1136/bmjopen-2015-009061
51. Hewlett, S., Almeida, C., Ambler, N., Blair, P., Choy, E., & Dures, E., Hammond, A., Hollingworth, W., Kadir, B., Kirwan, J., Plummer, Z., Rooke, C., Thorn, J., Turner, N. & Pollock (2019). Reducing arthritis fatigue impact: Two-year randomised controlled trial of cognitive behavioural approaches by rheumatology teams (RAFT). *Annals of the Rheumatic Diseases, 78*(4), 465–472. https://doi.org/10.1136/annrheumdis-2018-214469
52. Higdon, J. F. (1990). Expressive therapy in conjunction with psychotherapy in the treatment of persons with multiple personality disorder. *American Journal of Occupational Therapy, 44*(11), 991–993. doi: 10.5014/ajot.44.11.991
53. Hildebrand, M. W. (2015). Effectiveness of interventions for adults with psychological or emotional impairment after stroke: An evidence–based review. *American Journal of Occupational Therapy, 69*(1), 6901180050p1–6901180050p9. doi: 10.5014/ajot.2015.012054
54. Ho, E., & Siu, A. M. (2018). Occupational therapy practice in sleep management: A review of conceptual models and research evidence. *Occupational Therapy International*, 1–12. doi: 10.1155/2018/8637498
55. Hollon, T. H. (1972). The psychologist in the community general hospital. *Psychotherapy: Theory, Research & Practice, 9*(2), 180–184. doi: 10.1037/h0086743
56. Howells, J. G. (1975). Vector therapy in family psychiatry. *Child Psychiatry Quarterly, 8*(2), 13–22.
57. Hughes, J. (1989). Art in psychosocial occupational therapy: Guidelines for use and an art exercise battery*. Australian Occupational Therapy Journal, 36*(1), 14–23. doi: 10.1111/j.1440-1630.1989.tb01635.x
58. Jeffrey, L. (1973). Child psychiatry – The need for occupational therapy. *British Journal of Occupational Therapy, 36*(8), 429–437. doi: 10.1177/030802267303600802
59. Job, T., Broom, W., & Habermehl, F. (1997). Coming out! Time to acknowledge the importance of counselling skills in occupational therapy. *British Journal of Occupational Therapy, 60*(8), 357–358. doi: 10.1177/030802269706000808
60. Johnston, M. T . (1987). Occupational therapists and the teaching of cognitive behavioral skills. *Occupational Therapy in Mental Health, 7*(3), 69–81. https://doi.org/10.1300/J004v07n03_05
61. Keller, G. (2001). Body centered art activity: Development of lexithymic body awareness in occupational therapy and professional training*. Canadian Art Therapy Association Journal, 14*(2), 29–43. doi: 10.1080/08322473.2001.11434754
62. Körlin, D., Nybäck, H., & Goldberg, F. S. (2000). Creative arts groups in psychiatric care development and evaluation of a therapeutic alternative. *Nordic Journal of Psychiatry, 54*(5), 333– 340. doi: 10.1080/080394800457165
63. Levens, M. (1986). The psychodynamics of activity. *British Journal of Occupational Therapy, 49*(3), 87–89. doi: 10.1177/030802268604900310
64. Lewis, J.M. (1962). Therapeutic responsibilities. *American Journal of Occupational Therapy, 16*(6), 282–285.
65. Lichtenberg, P. A., Kimbarow, M. L., Morris, P., & Vangel Jr, S. J. (1996). Behavioral treatment of depression in predominantly African-American medical patients. *Clinical Gerontologist, 17*(2), 15–33. doi: 10.1300/J018v17n02_03
66. MacLiam, F. (2015). Cognitive behavioural psychotherapy graduates in Ireland: a follow-up survey of graduates from an Irish university*. Irish Journal of Psychological Medicine, 32*(2), 187– 195.
67. Malcolm, M. (1975). Art as a projective technique. *British Journal of Occupational Therapy, 38*(7), 147–148. doi: 10.1017/ipm.2014.51
68. Maree, M. (2007). The utilisation of Gestalt Play Therapy in occupational therapy intervention with traumatised children (Publication No. 0669133) [Master's thesis, University of South Africa]. *ProQuest Dissertations Publishing.*
69. Martin, J. E. (1990). Bulimia: A review of the medical, behavioural and psychodynamic models of treatment. *British Journal of Occupational Therapy, 53*(12), 495–500. doi: 10.1177/030802269005301204
70. Martin, S. (2007). How nurses and other healthcare professionals training in cognitive behavioural therapy experience the challenge of applying new learning in their clinical practice: a grounded theory study (Publication No. 13840796) [Doctoral dissertation, University of Abertay Dundee]. *ProQuest Dissertations Publishing*.
71. McKie, S., & Mathai, J. (1987). Combined treatment approach in the management of a 12-year-old boy presenting with disturbance of emotions. *British Journal of Occupational Therapy, 50*(3), 98–100. doi: 10.1177/030802268705000311
72. McLean, H. (1975). An encounter: Occupational therapy and psychodrama. *British Journal of Occupational Therapy, 38*(8), 168–171. doi: 10.1177/030802267503800806
73. Michael, L. (2019). Reviving nostalgia for an era of practice: An illustration of the therapeutic use of projective methods/media in occupational therapy. *Occupational Therapy in Mental Health, 35*(1), 1–20. doi: 10.1080/0164212X.2018.1538844
74. Miller, K., & Matthews, D. (1988). Setting up a ward-based group therapy programme for psychiatric inpatients. *British Journal of Occupational Therapy, 51*(1), 22–24. doi: 10.1177/030802268805100110
75. Moffatt, F., Mohr, C., & Ames, D. (1995). A group therapy programme for depressed and anxious elderly inpatients. *International Journal of Geriatric Psychiatry, 10*(1), 37–40. doi: 10.1002/gps.930100108
76. Moll, S. E., Tryssenaar, J., Good, C. R., & Detwiler, L. M. (2013). Psychotherapy: A profile of current occupational therapy practice in Ontario. *Canadian Journal of Occupational Therapy, 80*(5), 328–336. doi: 10.1177/0008417413515849
77. Moro, C. D. (2007). A comprehensive literature review defining self-mutilation and occupational therapy intervention approaches: Dialectical behavior therapy and sensory integration. *Occupational Therapy in Mental Health, 23*(1), 55–67. doi: 10.1300/J004v23n01_04
78. Murphy, S. L., Janevic, M. R., Lee, P., & Williams, D. A. (2018). Occupational therapist–delivered cognitive–behavioral therapy for knee osteoarthritis: A randomized pilot study. *American Journal of Occupational Therapy, 72*(5), 7205205040p1–7205205040p9. doi: 10.5014/ajot.2018.027870
79. Nalder, E., Marziali, E., Dawson, D. R., & Murphy, K. (2018). Delivering cognitive behavioural interventions in an internet-based healthcare delivery environment. *British Journal of Occupational Therapy, 81*(10), 591–600. doi: 10.1177/0308022618760786
80. Napoli, P. J., & Gold, B. (1947). Finger painting in an occupational therapy program. *American Journal of Occupational Therapy, 1*(6), 358–361.
81. Pol, M. C., Ter Riet, G., van Hartingsveldt, M., Kröse, B., & Buurman, B. M. (2019). Effectiveness of sensor monitoring in a rehabilitation programme for older patients after hip fracture: a three-arm stepped wedge randomised trial. *Age and Ageing, 48*(5), 650–657. https://doi.org/10.1093/ageing/afz074
82. Radnitz, A., Christopher, C., & Gurayah, T. (2019). Occupational therapy groups as a vehicle to address interpersonal relationship problems: mental health care users’ perceptions. *South African Journal of Occupational Therapy, 49*(2), 4–10. http://dx.doi.org/10.17159/2310-3833/2019/vol49n2a2
83. Read, H., Roush, S., & Downing, D. (2018). Early intervention in mental health for adolescents and young adults: A systematic review. *American Journal of Occupational Therapy, 72*(5), 7205190040p1-7205190040p8. doi: 10.5014/ajot.2018.033118
84. Reade, S., Hunter, H., & McMillan, I. R. (1999). Just playing… is it time wasted? *British Journal of Occupational Therapy, 62*(4), 157–162. doi: 10.1177/030802269906200405
85. Rothe, E. Q., Vega, B. J., Torres, R. M., Soler, S. M. C., & Molina, R. M. (2005). From kids and horses: Equine facilitated psychotherapy for children*. International Journal of Clinical and Health Psychology, 5*(2), 373–383.
86. Shannon, P. D., & Snortum, J. R. (1965). An activity group's role in intensive psychotherapy. *American Journal of Occupational Therapy, 19*(6), 344–347.
87. Short-DeGraff, M. A., & Engelmann, T. (1992). Activities for the treatment of combat-related post-traumatic stress disorder. *Occupational Therapy in Health Care, 8*(2–3), 27–47. doi: 10.1080/J003v08n02_02
88. Shortland-Jones, R., & Thompson, C. (2015). Mindfulness based interventions and cognitive behavioural therapy are shown to have similar effect in the short-term treatment of anxiety, depression and stress. *Australian Occupational Therapy Journal, 62*(2), 145–146. doi: 10.1111/1440-1630.12197
89. Simm, R. (2013). The role of occupational therapists in supporting psychological wellbeing after stroke using a solution-focused psychological approach to mood assessment. *British Journal of Occupational Therapy, 76*(11), 503–506. doi: 10.4276/030802213X13833255804630
90. Skinner, S. T. (1987). Multiple personality disorder: Occupational therapy intervention in acute care psychiatry. *Occupational Therapy in Mental Health, 7*(3), 93–108. doi: 10.1300/J004v07n03_07
91. Smallfield, S., & Molitor, W. L. (2018). Occupational therapy interventions addressing sleep for community-dwelling older adults: A systematic review. *American Journal of Occupational Therapy, 72*(4), 7204190030p1-7204190030p9. doi: 10.5014/ajot.2018.031211
92. Söchting, I., & Third, B. (2011). Behavioral group treatment for obsessive-compulsive disorder in adolescence: A pilot study. *International Journal of Group Psychotherapy, 61*(1), 84–97. doi: 10.1521/ijgp.2011.61.1.84
93. Sood, J. R., Cisek, E., Zimmerman, J., Zaleski, E. H., & Fillmore, H. H. (2003). Treatment of depressive symptoms during short-term rehabilitation: An attempted replication of the DOUR project. *Rehabilitation Psychology, 48*(1), 44–49. doi: 10.1037/0090-5550.48.1.44
94. Stein, F., & Tallant, B. K. (1988a). Applying the group process to psychiatric occupational therapy Part 1: Historical and current use. *Occupational Therapy in Mental Health, 8*(3), 9–28. doi: 10.1300/J004v08n03_02
95. Stein, F., & Tallant, B. K. (1988b). Applying the group process to psychiatric occupational therapy Part 2: A model for a therapeutic group in psychiatric occupational therapy. *Occupational Therapy in Mental Health, 8*(3), 29–51. doi: 10.1300/J004v08n03_03
96. Stoffel, V. C., & Moyers, P. A. (2004). An evidence-based and occupational perspective of interventions for persons with substance-use disorders. *American Journal of Occupational Therapy, 58*(5), 570–586. doi: 10.5014/ajot.58.5.570
97. Telford, R., & Ainscough, K. (1995). Non-directive play therapy and psychodynamic theory: Never the twain shall meet?. *British Journal of Occupational Therapy, 58*(5), 201–203. doi: 10.1177/030802269505800504
98. Thompson, M., & Blair, S. E. (1998). Creative arts in occupational therapy: ancient history or contemporary practise? *Occupational Therapy International, 5*(1), 48–64. doi: 10.1002/oti.67
99. Tokolahi, E., Em-Chhour, C., Barkwill, L., & Stanley, S. (2013). An occupation-based group for children with anxiety. *British Journal of Occupational Therapy, 76*(1), 31–36. doi: 10.4276/030802213X13576469254694
100. Torchalla, I., Killoran, J., Fisher, D., & Bahen, M. (2019). Trauma-focused treatment for individuals with posttraumatic stress disorder: The role of occupational therapy. *Occupational Therapy in Mental Health, 35*(4), 386–406. https://doi.org/10.1080/0164212X.2018.1510800
101. Vaughan, P. J., & Prechner, M. (1985). Occupation or therapy in psychiatric day care? *British Journal of Occupational Therapy, 48*(6), 169–171.
102. Wiskin, L. F. (1998). Cognitive-behavioral therapy: a psychoeducational treatment approach for the American worker with rheumatoid arthritis. *Work, 10*(1), 41–48. doi: 10.3233/WOR-1998-10107
103. Wittkower, E. D., & Azima, H. (1958). Dynamic aspects of occupational therapy. *A.M.A. Archives of Neurology & Psychiatry, 79*(6), 706–710. doi: 10.1001/archneurpsyc.1958.02340060104014
104. Ziesler, A. A. (1993). Art therapy–A meaningful part of cancer care. *Journal of Cancer Care*, 2, 107–111.

Type of research: As in the original study, half of the articles were non-empirical (*n = *52, 50%). The empirical articles (*n = *52; 50%) include quantitative (*n = *22, 21%), qualitative (*n = *17, 17%), reviews (*n = *11, 11%) and mixed methods (*n = *2, 2%) methodology.

Description of included studies: The articles were published between 1927 and 2020, with two articles published between 1927 and 1949 (2%), nine articles published between 1950 and 1972 (9%), nine published between 1973 and 1995 (9%) and 84 articles published between 1996 and 2020 (81%). 26 articles were published in the last ten years (25%). The articles were mostly from the United States and the United Kingdom (31–37%, *n = *32–38). Canadian researchers authored 9% of the total articles (*n = *9). Occupational therapy journals published 64% (*n = *67) of the articles in this review. Percentages have been rounded to whole numbers.

### Narrative Synthesis

**Figure 2.**
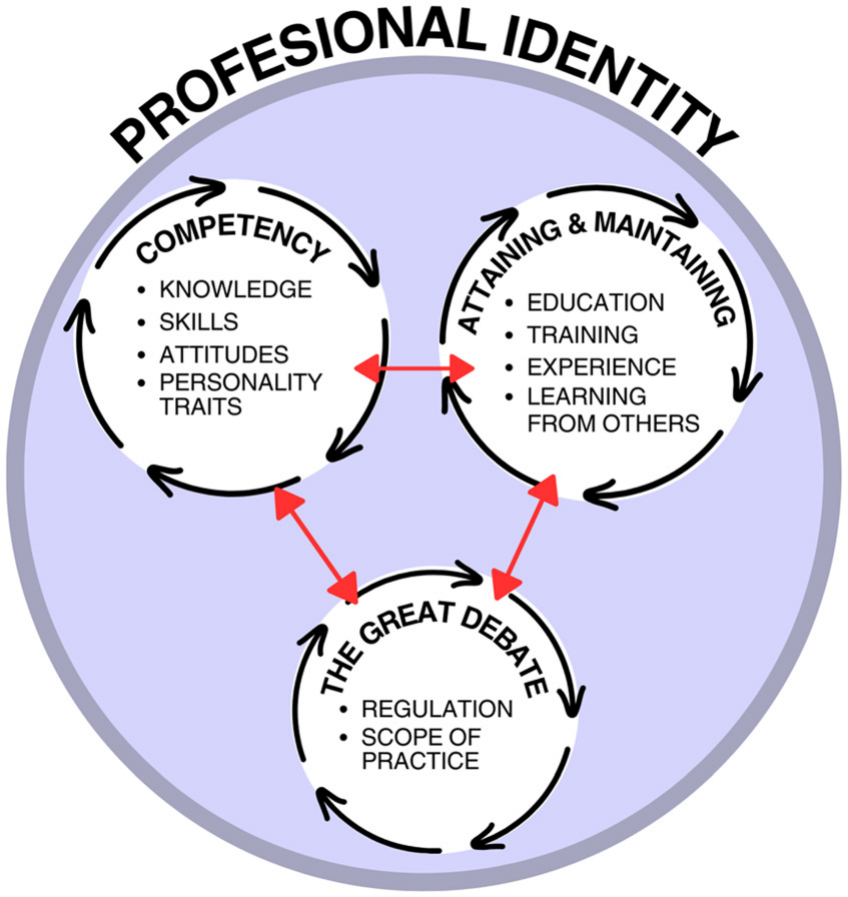
Visual representation of theme and subthemes.

One overall theme, *Professional Identity*, is further explained by three subthemes: *Competence*, *Attaining and Maintaining Competence*, and *The Great Debate* (Figure 2). A numbered list of the 104 included studies has been provided in Table 2. A list of the articles included in each theme and subtheme is provided in Table 3 and can be cross-referenced using Table 2.

**Table 3:** Theme Distribution

Theme	Subtheme		Quantity	%	Articles
Professional identity			8	7.69	1, 9, 14, 28, 57, 70, 76, 95
	Competency		23	22.12	1, 3, 6, 8, 9, 10, 11, 14, 25, 28, 34, 39, 41, 47, 52, 64, 76, 80, 89, 90, 98, 99, 101
		Knowledge	10	9.62	34, 39, 41, 52, 64, 76, 89, 90, 98, 99
		Skills	9	8.65	3, 8, 10, 14, 16, 25, 28, 52, 80
		Attitudes and personality traits	5	4.81	1, 9, 11, 47, 101
	Attaining and maintaining competency		91	87.5	1, 2, 3, 4, 5, 6, 7, 8, 9, 11, 12, 13, 14, 15, 17, 18, 19, 20, 21, 22, 23, 24, 26, 28, 29, 30, 31, 32, 33, 35, 36, 37, 38, 39, 40, 42, 43, 44, 45, 46, 47, 48, 49, 50, 51, 53, 54, 55, 56, 57, 58, 59, 60, 62, 63, 64, 65, 66, 67, 68, 69, 70, 71, 72, 73, 74, 75, 76, 78, 79, 80, 81, 83, 84, 85, 86, 87, 88, 91, 92, 93, 94, 95, 96, 97, 98, 100, 101, 102, 103, 104.
		Entry-to-practice education	21	20.19	7, 12, 24, 29, 30, 48, 53, 58, 68, 76, 91, 87, 83, 103, 17, 5, 102, 32, 63, 72, 97
		Continuing education and training	70	67.31	1, 2, 3, 4, 6, 8, 9, 12, 13, 14, 15, 18, 19, 21, 22, 23, 26, 28, 29, 31, 33, 35, 36, 37, 38, 40, 42, 43, 44, 45, 46, 47, 48, 49, 50, 51, 53, 54, 55, 56, 57, 58, 59, 62, 65, 66, 68, 70, 71, 73, 75, 76, 78, 79, 80, 81, 84, 85, 86, 88, 92, 93, 94, 96, 97, 98, 100, 101, 103, 104
		Experience	17	16.35	1, 3, 8, 9, 11, 29, 37, 39, 60, 50, 57, 67, 69, 76, 88, 98, 100
		Learning from others	21	20.19	5, 13, 15, 20, 21, 29, 30, 51, 55, 57, 64, 65, 66, 70, 74, 76, 80, 84, 93, 97, 103
	The great debate		16	15.38	3, 5, 14, 16, 20, 27, 33, 41, 53, 65, 73, 76, 77, 82, 84, 89
		Regulation	12	11.54	16, 20, 27, 33, 41, 53, 65, 73, 76, 77, 82, 89
		Scope of practice	7	6.73	3, 5, 14, 33, 41, 76, 84

#### Professional Identity

*Professional identity* refers to occupational therapists’ sense of belonging to their profession. The integration of psychotherapy into occupational therapy practice can present challenges to professional identity. Integrating psychotherapy into practice can challenge this identity, especially if occupation-based approaches are not used (Clarke, 1999). This theme included 8% of the articles (*n = *8) and ranged from 1971 to 2019.

In a survey of Ontario occupational therapists, most of the participants reported beginner to moderate perceived competence in psychotherapeutic approaches (Moll et al., 2013). Many occupational therapists report hesitancy in adopting psychotherapeutic interventions due to a lack of formal training, having doubts about their ability to effectively deliver psychotherapy (Hughes, 1989). Even after occupational therapists undertake continuing education, some continue to feel hesitant to practice psychotherapy (Hughes, 1989). Concerns about managerial approval and perceptions of colleagues also contribute to role dissonance, with occupational therapists fearing that incorporating psychotherapy could be seen as overstepping professional boundaries (Martin, 2007).

The ambivalence about whether psychotherapy falls within the scope of occupational therapy (Marshall et al., 2022; Moll et al., 2013), coupled with concerns about competence, can contribute to role confusion and role dissonance (Burton, 1954; Martin, 2007). These issues contribute to a sense of uncertainty and conflict regarding one's professional role (Martin, 2007), which can ultimately threaten professional identity.

##### Competence

*Competence* refers to the knowledge, skills, and attitudes (College of Occupational Therapists of British Columbia, n.d.; Kaslow, 2004) that underlie effective psychotherapy provision. This subtheme included 22% (*n = *23) of the articles, spanning from 1927 to 2017, and covers general competencies that have been reported across time rather than an exhaustive list of specific competencies. If a practitioner's knowledge is limited, treatment decisions may also be limited resulting in treatment that is not effective (Moll et al., 2013). Authors discussed the general areas of knowledge essential for psychotherapy competence: the psychology of individuals (German, 1964; Lewis, 1962) and groups (German, 1964; Levens, 1986; Lewis, 1962), psychological disorders (Skinner, 1987), and treatment modalities (Thompson & Blair, 1998). Several authors report that most Canadian occupational therapy students learn foundational psychotherapy knowledge, including establishing and maintaining a therapeutic relationship (Colman, 1975; Higdon, 1990; Read et al., 2018; Short-DeGraff & Engelmann, 1992; Simm, 2013; Tokolahi et al., 2013).

Five articles discuss general skills required by occupational therapists practicing psychotherapy: setting appropriate boundaries (Alers, 2008), non-judgemental listening (Broadbent, 1985; Carpenter, 2014), responding and synthesizing information (Broadbent, 1985; Carpenter, 2014), therapist self-disclosure (Broadbent, 1985), immediacy (Broadbent, 1985), confrontation (Broadbent, 1985), problem-solving (Carpenter, 2014), developing collaborative treatment plans (Carpenter, 2014), and being able to assess one's own competence (Stein & Tallant, 1988b).

Attitudes and personality traits may enhance one's competence, and according to some authors, may be more important than the skills of the therapist (Haviland, 1927; Vaughan & Prechner, 1985). Four articles mention specific personality traits and attitudes that enhance psychotherapy competence: tenacity, adaptability, commitment, trustworthiness (Alers, 2008), genuineness (Haviland, 1927), confidence (Alers, 2008; Burton, 1954), and having an empathetic and empowering attitude (Alers, 2008; Green et al., 2008; Haviland, 1927).

##### Attaining and Maintaining Competence

This subtheme (88%; *n = *91) focuses on developing and maintaining psychotherapy competence through education, training, and experience. Rather than a specific and sequential pathway to psychotherapy competence, this subtheme includes information about isolated methods of attaining and maintaining psychotherapy competence that has been reported between 1927 and 2020 and is not exhaustive.

Entry-to-practice education introduces psychotherapy competence through coursework on therapeutic dynamics, psychological functioning, and group therapy (Colman, 1975; Gardiner et al., 2017; Hildebrand, 2015; McLean, 1975; Telford & Ainscough, 1995). Hayner (1999) and Moll and colleagues (2013) described student therapists needing more psychotherapy education within the entry-to-practice education program to feel confident providing psychotherapy.

Educational topics suggested in the literature to include in entry to practice education include psychotherapeutic approaches for children (Chan et al., 2017; Maree, 2007), behavioural activation (Brick et al., 2020), complex trauma (Maree, 2007), CBT for sleep (Smallfield & Molitor, 2018), expressive therapies (Froehlich, 1992), and the development of psychological goals (Hayner, 1999).

Continuing education and training refers to additional psychotherapy training post-entry-to-practice education, including formal training courses, supervision, communities of practice or study groups. Two articles mention the potential for therapists to improve their psychotherapeutic knowledge and skills when they engage in continuing education (Broadbent, 1985; Clarke, 1999). Nine articles describe the need for therapists to take additional psychotherapy continuing education, to use psychotherapy effectively in their practice (Burton, 1954; Clarke, 1999; Cline & Rouzer, 1971; Craik et al., 1998; Hildebrand, 2015; Job et al., 1997; Stein & Tallant, 1988a; Wittkower & Azima, 1958); however, they are not specific about the type and amount of education needed. Moll and colleagues (2013) discuss while it may not be feasible to be trained in all psychotherapeutic approaches, it is important to have a general understanding of various approaches in order to select the most appropriate approach for the client. Some occupational therapists are concerned about their competence and hesitant to practice psychotherapy without continuing education (Hughes, 1989).

Barriers to occupational therapists pursuing additional training include the cost and time required (MacLiam, 2015). Even after completing continuing education, therapists have difficulty practicing psychotherapy (Gagnon, 1996; MacLiam, 2015) because of the demands to complete other tasks in their practice (MacLiam, 2015; Martin, 2007). Therapists also express concern about colleagues’ perceptions of occupational therapists providing psychotherapy (Martin, 2007).

Learning from others is how occupational therapists increase their competence through supervision, communities of practice, professional study groups and informal interactions. Thirteen articles recommended that supervision be available to those using psychotherapy, to help develop skills, implement training, determine future training needs, and ultimately maintain competence (Cline & Rouzer, 1971; Cooper & Davis, 2016; Copley et al., 1987; Hewlett et al., 2015; MacLiam, 2015; Miller & Matthews, 1988; Moll et al., 2013; Napoli & Gold, 1947; Reade et al., 1999; Telford & Ainscough, 1995; Ziesler, 1993) and manage challenges (Miller & Matthews, 1988). Froehlich (1992) and Hughes (1989) suggest that supervision is especially important for novice therapists. Martin (2007) and Moll and colleagues (2013) suggest that therapist supervision needs are unmet.

Online or in person, communities of practice (Moll et al., 2013) webinars (Fraser et al., 2019), professional study groups (Fraser et al., 2019), and discussing and observing psychotherapy with experienced therapists improve therapist psychotherapy skills and confidence (Froehlich, 1992).

Experience through the “doing” of psychotherapy, as the therapist or client, improves psychotherapy knowledge and skills (Broadbent, 1985; Champernowne, 1952; Johnston, 1987; Shortland-Jones & Thompson, 2015). Having personal therapy experience helps the therapist gain an understanding of psychotherapy techniques and modalities and insight into the therapy process (Champernowne, 1952’ Johnston, 1987).

Experienced occupational therapists are more likely to feel competent delivering psychotherapy (Alers, 2008; Moll et al., 2013) because competence improves as therapists work with more clients, becoming more skilled through “trial and error” (Burton, 1954, p. 79). Experienced occupational therapists also experience lower client dropout rates from psychotherapy (Martin, 1990). Furthermore, experienced occupational therapists use their psychotherapy experience to increase competence in others through training and supervision (Green et al., 2005; Reade et al., 1999).

##### The Great Debate

Occupational therapists have varying perspectives about if and how psychotherapy fits into occupational therapy’s scope of practice. This subtheme includes 15% (*n = *16) of the articles in this review, ranging from 1971 to 2019.

Professional regulation certifies that individuals have the education, experience, and training required to be a competent professional (Michael, 2019; Moll et al., 2013); however, it is the therapist's responsibility to attain and maintain professional competence. In Ontario, COTO implements psychotherapy as a controlled act for occupational therapists and provides standards of practice for occupational therapists providing psychotherapy to support the self-assessment of their competence (Moll et al., 2013). However, across Canada, psychotherapy regulation varies widely between provinces (Moll et al., 2013), and such supporting documents are not available in other provinces, contributing to uncertainty around the inclusion of psychotherapy within occupational therapy (Moll et al., 2013; Reade et al., 1999).

Typically, occupational therapy's scope of practice is not specified by treatment modality, leading to debate about whether psychotherapy is part of occupational therapy (Moll et al., 2013; Reade et al., 1999). In a survey conducted by Moll and colleagues (2013), some Ontario therapists view psychotherapy as “separate” (p. 334) from their practice as an occupational therapist, while others view their practice of psychotherapy and occupational therapy as integrated and inseparable. On the other hand, Birkholtz and Blair (2001) and Clarke (1999) differentiate occupational therapy psychotherapy by stating that the use of activity or occupation is integral to occupational therapy psychotherapy (Birkholtz & Blair, 2001).

## Discussion

This is the first study to review psychotherapy competence in occupational therapy literature, analyzing 52.5% (*n = *104) of articles from Marshall et al. (2022). Most studies were published after 2000 (*n = *55, 53%), and 50% were non-empirical. The original and current datasets had similar distributions of articles by publication year, research type, and country of origin.

While the articles included in this dataset span several decades, dating back to 1927, each theme also includes recent articles, that is, 2017–2022. For example, the articles that were themed around *The Great Debate* spanned from 1971 to 2019, the articles on *Competence* spanned from 1927 to 2017, and the articles included in *Attaining and Maintaining Competence* spanned from 1927 to 2020. The issue of *Professional Identity* among occupational therapists who practice psychotherapy is a concern that continues today (Martin, 2007; Moll et al., 2020a, 2020b). The continually evolving regulation of psychotherapy (Marshall et al., 2022; Moll et al., 2013) combined with the persistent identity-related challenges faced by occupational therapists (Holland et al., 2013; Souto-Gómez et al., 2023; Walder et al., 2022) underscore the enduring importance of the findings of this study.

The narrative synthesis highlights the role of *Professional Identity* in the psychotherapy competence of occupational therapists. Professional identity refers to an individual's sense of belonging to a professional group (Abreu, 2006; Adams et al., 2006), which evolves as one internalizes shared knowledge, values, and skills (Schein, 1978; Souto-Gómez et al., 2023).

Figure 2 illustrates the dynamic relationship between *Professional Identity* and the three subthemes: *Competence, Attaining and Maintaining Competence,* and *The Great Debate.* The three subthemes are dynamic and continually influence each other while contributing to an evolving professional identity (Figure 2). This analysis suggests that psychotherapy competence develops through education, learning from others, and experience. However, the literature included in this review lacks a clear pathway for attaining competence, contributing to uncertainty for occupational therapists.

Psychotherapy competence within occupational therapy includes knowledge, skills, attitudes, and related personality traits (*Competence*). The definition of professional identity (Adams et al., 2006; Hooper, 2008; Schein, 1978) encompasses the same elements as psychotherapy competence outlined in this review.

The subtheme that describes methods of *Attaining and Maintaining Competence*, overlaps with the same methods that are used to develop professional identity (Walder et al., 2022). Education improves occupational therapists’ psychotherapy knowledge and skills, therefore improving competence, confidence, and professional identity (Holland et al., 2013; Souto-Gómez et al., 2023; Walder et al., 2022). Therapists who are competent can assist in increasing the competence of other therapists through training (Green et al., 2005), thereby contributing to the professional identity of both occupational therapists (Walder et al., 2022). The authors of approximately 20% of the articles in this study reported occupational therapists needing more psychotherapy education in the entry-to-practice education program and continuing education to be competent in providing psychotherapy. Occupational therapy students graduate from their entry-to-practice education program unclear about their role and lack confidence in their psychotherapy competence, how to attain competence, and question if psychotherapy is part of their scope of practice. This leaves new therapists who want to practice psychotherapy questioning their professional identity (Souto-Gomez et al., 2023).

Entry-to-practice education aims to clarify professional identity, but research shows that identity only stabilizes after five years of practice (Souto-Gómez et al., 2023). Given that new therapists in years one to five experience an underdeveloped professional identity, there is a need to support these therapists who wish to practice psychotherapy (Souto-Gómez et al., 2023).

*The Great Debate* subtheme reflects conflicting perspectives on how psychotherapy fits within the scope of occupational therapy. Other professions who practice psychotherapy do not have this same debate because psychotherapy is explicitly mentioned in their governing professional documents (e.g., Canadian Association of Social Workers, 2020; Canadian Medical Association, 2019; Manitoba College of Social Workers, n.d.; Psychological Association of Manitoba, n.d.). Occupational therapy education programs across Canada aim to graduate generalist practitioners, equipped with broad competencies, versus competencies in specific treatment modalities (CAOT, 2024a, 2024b; Committee on University Fieldwork Education & Association of Canadian Occupational Therapy University Programs, 2024; Donnelly et al., 2023; Freeman et al., 2014). Accordingly, the regulators across Canada, with the exception of Ontario, do not specify competencies for specific interventions like psychotherapy. Instead, regulators focus on competence related to broader knowledge, skills, and attitudes required for all interventions. While graduating as a generalist equips occupational therapists to address diverse client needs in various settings, occupational therapy positions increasingly require specialized knowledge and skills, such as those needed for psychotherapy (Freeman et al., 2014).

In Ontario, occupational therapists registering to practice psychotherapy must complete a mandated supervisory period (College of Registered Psychotherapists of Ontario, 2025) similar to psychotherapists from psychology and psychiatry (Government of Manitoba, 2006; University of Manitoba, n.d.). Quebec occupational therapists practicing psychotherapy are regulated by the Ordre Des Psychologues Du Québec and are also required to receive supervision (Ordre Des Psychologues Du Québec, 2025). This supervision requirement aligns with the World Federation of Occupational Therapists’ (WFOT, 2014) recommendation that occupational therapists seeking specialization in any practice area participate in supervision as part of developing advanced competencies. Other than Ontario and Quebec, other Canadian provinces do not require psychotherapy supervision, which may limit occupational therapists’ ability to acquire specialized skills needed for psychotherapy post-graduation (WFOT, 2014).

The inconsistency of supervisory requirements across provinces may exacerbate the conflicting perspectives on whether psychotherapy aligns with the scope of occupational therapy. Without this structured support for developing advanced competencies, occupational therapists may struggle to integrate psychotherapy into their practice, further complicating professional identity. Even in Ontario, where psychotherapy is regulated and supervision is required, Vesely and colleagues (2023) noted that the inclusion of multiple professions authorized to practice psychotherapy can create confusion about professional roles and boundaries, contributing to challenges in professional identity.

Additionally, professional identity is challenged because there is a lack of consensus around the definition of psychotherapy (Marshall et al., 2022) adding to the complexity of what it means to be competent. This lack of consensus about the definition of psychotherapy is common in other professions that provide psychotherapy such as psychology and social work (Moroz et al., 2020) creating murkiness to the task of discussing psychotherapy competence.

Occupational therapy regulation across Canada determines the knowledge, skills, and attitudes required to attain professional competence (*Competence*). However, the education, supervision, and experience required to maintain competence are determined by an occupational therapist's self-assessment. The self-assessment is used by the therapist to determine their learning needs to *attain and maintain competence* (CAOT, 2012; COTM, 2010). CAOT recently released a practice document for psychotherapy, which supports the role of occupational therapists in psychotherapy while highlighting the varied provincial regulations and ongoing debate (CAOT, 2024c). However, this resource does not outline a pathway to psychotherapy competence for occupational therapists.

When professional identity is unclear such as in the case of psychotherapy competence (Moll et al., 2013), practitioners may not understand what psychotherapy competence is or how to attain it. This disconnect results in an unclear professional identity that may influence the quality of the services that are provided (Takashima & Saeki, 2019) including psychotherapy. Therapists confident in their scope of practice tend to have a more developed professional identity (Holland et al., 2012), therefore occupational therapists who are confident that psychotherapy is or is not part of their scope of practice have a more developed professional identity than therapists who are unsure if psychotherapy is part of their role or scope of practice.

The three subthemes contribute to an evolving occupational therapy professional identity that is challenged when occupational therapists practice psychotherapy. This challenge is heightened for newly graduated therapists transitioning to practice, as they often experience role confusion and lack a clear professional identity (Souto-Gómez et al., 2023).

To support the competence and professional identity of occupational therapists providing psychotherapy, it is important to explore what occupational therapists need to feel confident and competent in providing this service. For those practicing psychotherapy, building occupational therapists’ confidence may nurture professional identity.

Several authors (Birkholtz & Blair, 2001; Clarke, 1999; Marshall et al., 2022; Moll et al., 2020a, 2020b) call on occupational therapists to practice occupation-based psychotherapy. Placing occupation as the core of psychotherapy practice may support professional identity development. Cooper (2012), in her Muriel Driver lecture, encouraged the profession to look outward. If we are to continue growing as a profession, we must make clear the unique contributions occupational therapists can make with “occupation [at] the core of [everything we do]” (Cooper, 2012, p. 203).

### Limitations of the Study Design

Secondary data analysis is limited by how well the secondary research question fits within the parameters of the original study and the quality of the original data collection (Castle, 2003; Cheng & Phillips, 2014; Heaton, 2011). This study mitigated these limitations by having two researchers involved throughout the process, one of whom was part of the original study. We assumed the original study captured all relevant research in psychotherapy within occupational therapy.

Another limitation is that some of the articles in this review date back several decades, which may lead readers to question the relevance of psychotherapy competence of occupational therapists in today's context. This limitation was addressed by noting the span of years of the articles for each theme demonstrating the persistence of the themes despite the passage of time. The fact that there are recent articles for each theme demonstrates that the themes have an enduring importance.

Author positionality: Both authors of this research are occupational therapists with a keen interest in psychotherapy within occupational therapy. This bias was addressed through discussions with others inside and outside of the occupational therapy profession.

The articles included in this scoping review were not assessed for quality, as the original study assessed the evidence to be poor with almost half of the articles being non-empirical.

## Conclusion

The scope of existing literature on psychotherapy competence within occupational therapy includes a description of what psychotherapy competence is, how it is attained and maintained, the debate surrounding this topic, and the impact on professional identity. The reciprocal relationship between professional identity and psychotherapy competence, particularly in the first five years of practice, is of particular interest. This review highlights the need to support the professional identity of early-career occupational therapists practicing psychotherapy. This review also highlights the lack of clear pathways to establishing psychotherapy competence, which impacts professional identity. Future research should examine the need for clear pathways to psychotherapy competence and how they can support occupational therapists’ unique role in occupation-based psychotherapy.

As mental health concerns continue to rise in Canada, occupational therapists must promote their unique occupation-based approach to psychotherapy (Marshall et al., 2022) which connects meaningful activities with health and well-being, empowering clients through “doing, being, and becoming” (Wilcock, 1999). This holistic approach fosters resilience and mental health, emphasizing the critical role of occupation in well-being.

## Key Messages

- There is a need to assess what occupational therapists need to be confident and competent in providing psychotherapy.
- Clear pathways to attaining psychotherapy competence are needed to strengthen professional identity and enhance competence, given their reciprocal relationship.
- Occupational therapists with five years or less of practice are at risk of a weakened professional identity, which can affect competence; they require specialized support.
